# Invasive *Geotrichum klebahnii* fungal infection: A case report

**DOI:** 10.1099/acmi.0.000287

**Published:** 2021-11-30

**Authors:** Vhudzani Tshisevhe, Barend Mitton, Lebogang Skosana

**Affiliations:** ^1^​ Lancet Laboratories, Rustenburg, South Africa; ^2^​ Department of Medical Microbiology, University of Pretoria, Pretoria, South Africa; ^3^​ Department of Medical Microbiology, Tshwane Academic Division, National Health Laboratory Service, Pretoria, South Africa

**Keywords:** Geotrichosis, *Geotrichum klebahnii*, *Geotrichum* species, Invasive fungal infection, rare fungal infection

## Abstract

Geotrichosis is a world-wide mycosis caused by *Geotrichum* species. We report a rare case of an invasive cutaneous infection by *Geotrichum klebahnii* in a female patient with undiagnosed diabetes mellitus. The patient presented with right facial swelling not responding to antibiotics and could not recall trauma to the site of the lesion. Histological examination showed fungal hyphae invading salivary glands and bony tissues, and *G. klebahnii* was isolated from the culture of biopsy material. Matrix-assisted laser desorption/ionization-time of flight (MALDI-TOF) mass spectrometry (MS) confirmed the fungal species. Broth microdilution showed low minimum inhibitory concentrations (MICs) for itraconazole, posaconazole, voriconazole and amphotericin B. Treatment with sequential administration of intravenous amphotericin B with voriconazole followed by itraconazole led to the resolution of the lesion.

## Introduction

*Geotrichum* is a genus of environmental yeast-like fungi with worldwide distribution found in soil, water, air, and commonly as part of the normal human flora [1]. These fungi are members of *Endomycetaceae* family from Saccharomycetales class [2]. *Geotrichum candidum* is an ubiquitous fungus known to colonize the human skin, respiratory system and gastrointestinal tract [3–5]. It is also the commonest causative agent of geotrichosis [6–8]. *Geotrichum klebahnii* is a rarely described fungus originally isolated from mandarin peel [9].

*Geotrichum* species are considered to be of low virulence, but they have been found to cause diseases [3, 10–13]. Geotrichosis affects mainly patients who have underlying immunocompromising conditions such as neoplasms, diabetes mellitus, renal impairment, organ transplant, burns and human immunodeficiency virus (HIV) infection [14, 15]. Traumatic inoculation can also lead to the development of disease in an apparently healthy and immunocompetent individual [14, 16].

Disease presentation is highly variable largely due to individual host predisposition. Pulmonary geotrichosis is the most frequently reported form of the disease, but bronchial, oral, vaginal, cutaneous and alimentary infections have also been reported [14, 17]. Patient recovery largely depends on risk factors and management of the underlying condition but *Geotrichum* fungemia is associated with poor prognosis, especially in patients with prolonged neutropenia as a complication of haematological malignancy [18, 19].

We present a case of invasive geotrichosis involving a patient who was not known to have prior predisposition and an unusual *Geotrichum species.*

## Case presentation

A female in her thirties, with no known chronic medical conditions, presented to hospital with a 3 month history of right maxillary swelling. Prior to this presentation, the patient was treated with amoxicillin-clavulanic acid for 2 weeks and ciprofloxacin for ten days with no clinical improvement. The choice of antimicrobial agents was based on the community general practitioner’s assumption that the patient had a bacterial infection. The swelling was initially not painful but pain started and increased with swelling after 6 weeks since initial presentation. A fungal infection was considered at the community clinic and the patient was initiated on fluconazole 2 weeks before the current presentation. The patient reported worsening of the condition with an associated visual disturbance as the swelling increased. She reported no recollection of trauma. Physical examination of the patient revealed right orbital and maxillary swelling with mild eye proptosis. The lesion measuring 12×6 cm was surrounded by mild erythema and was tender on palpation. Fundoscopy was uneventful. Serum galactomannan tested negative. The patient tested negative for HIV. Exploration of the maxillary antrum revealed a periorbital mass extending into the maxillary sinus. The mass was excised and submitted to the laboratory for culture and histological assessment.

## Laboratory investigations

**Fig. 1.**
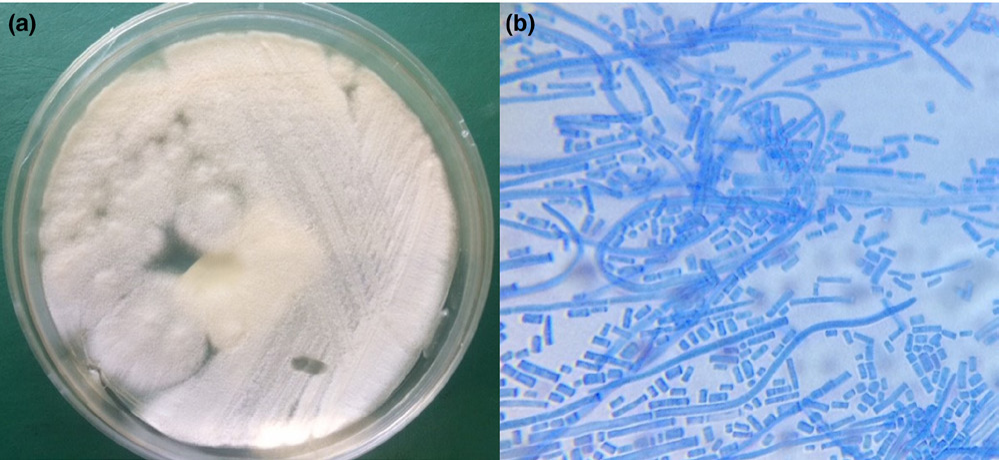
(a) Tissue culture showing growth of flat, off-white to cream, dry and finely suede-like colonies after 24 h of incubation on Sabouraud dextrose agar. (b) Lactophenol cotton blue stain microscopy at 1000x magnification showing hyaline, septate hyphae which break up into chains of hyaline, smooth, one-celled arthroconidia.

The culture of the tissue (Fig. 1a) showed growth of flat, off-white to cream, dry and finely suede-like colonies after 24 h of incubation at 25 °C on Sabouraud dextrose agar. There was no pigment production. Lactophenol cotton blue stain (Fig. 1b) revealed hyaline, septate hyphae that break up into chains of hyaline, smooth, one-celled arthroconidia. There was no blastoconidia observed. Identification of the organism was performed by assimilation tests performed with both API 20C (bioMérieux, Marcy l’Etoile, France) and the automated VITEK 2 System (bioMérieux, Marcy-l'Etoile, France) using YST card, and Biotyper Matrix-assisted laser desorption/ionization-time of flight (MALDI-TOF) mass spectrometry (MS) (MALDI Biotyper, Bruker Scientific Instruments, Billerica, MA, USA) which identified the organism as *G. klebahnii*. The cultured isolate assimilated glucose, glycerol, xylose and galactose. The broth microdilution susceptibility testing (Sensititre YeastOne, Thermo Fisher Scientific, Waltham, MA, USA) showed minimum inhibitory concentration (MIC) values of 16 µg ml^−1^ for fluconazole, 0.12 µg ml^−1^ for itraconazole, 0.5 µg ml^−1^ for posaconazole, 8 µg ml^−1^ 5-flucytosine, 0.06 µg ml^−1^ for voriconazole and 0.12 µg ml^−1^ for amphotericin B. Mycobacterial and bacterial culture did not yield any microorganism growth.

**Fig. 2.**
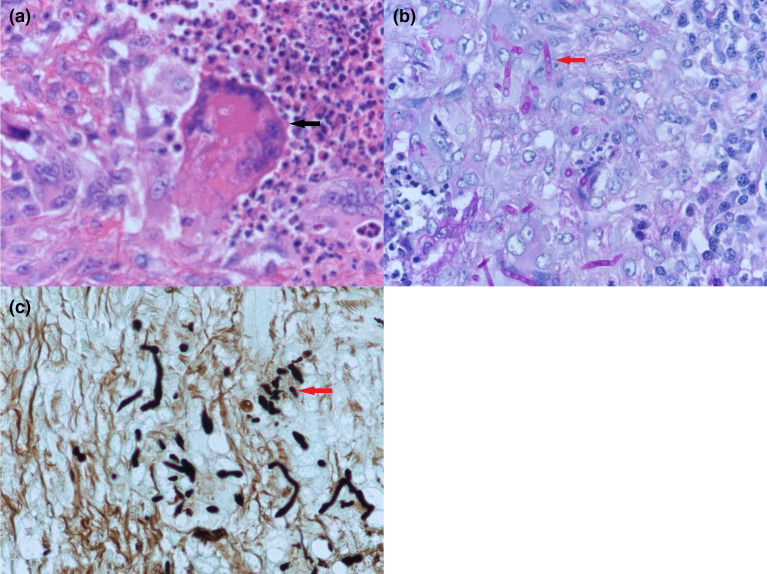
(a) Hematoxylin and eosin stain microscopy of tissue biopsy at 400x magnification showing a granuloma with central neutrophilic micro-abscess formation. (b) Periodic acid-Schiff stain microscopy of tissue biopsy at 400x magnification highlighting arthroconidia. (c) Grocott-Gomori’s silver stain microscopy of tissue biopsy at 400x magnification showing fungal elements.

Histological examination of the tissue submitted showed the presence of polypoid lesions lined by respiratory epithelium. Some normal minor salivary glands as well as bony tissue are identified. The lesions consist of dense granulomatous inflammation. Some of the granulomas (Fig. 2a) show central neutrophilic micro-abscess formation. The intervening fibrous stroma contains lymphocytes and plasma cells. The presence of fungal hyphae is noted (Fig. 2a). The fungal hyphae show marked fragmentation (arthroconidia) with no branching seen. Periodic acid Schiff (PAS) (Fig. 2b) and Grocott-Gomori’s Methenamine Silver (Fig. 2c) stains highlighted the arthroconidia. Ziehl-Neelsen stain showed no acid-fast bacilli.

**Fig. 3.**
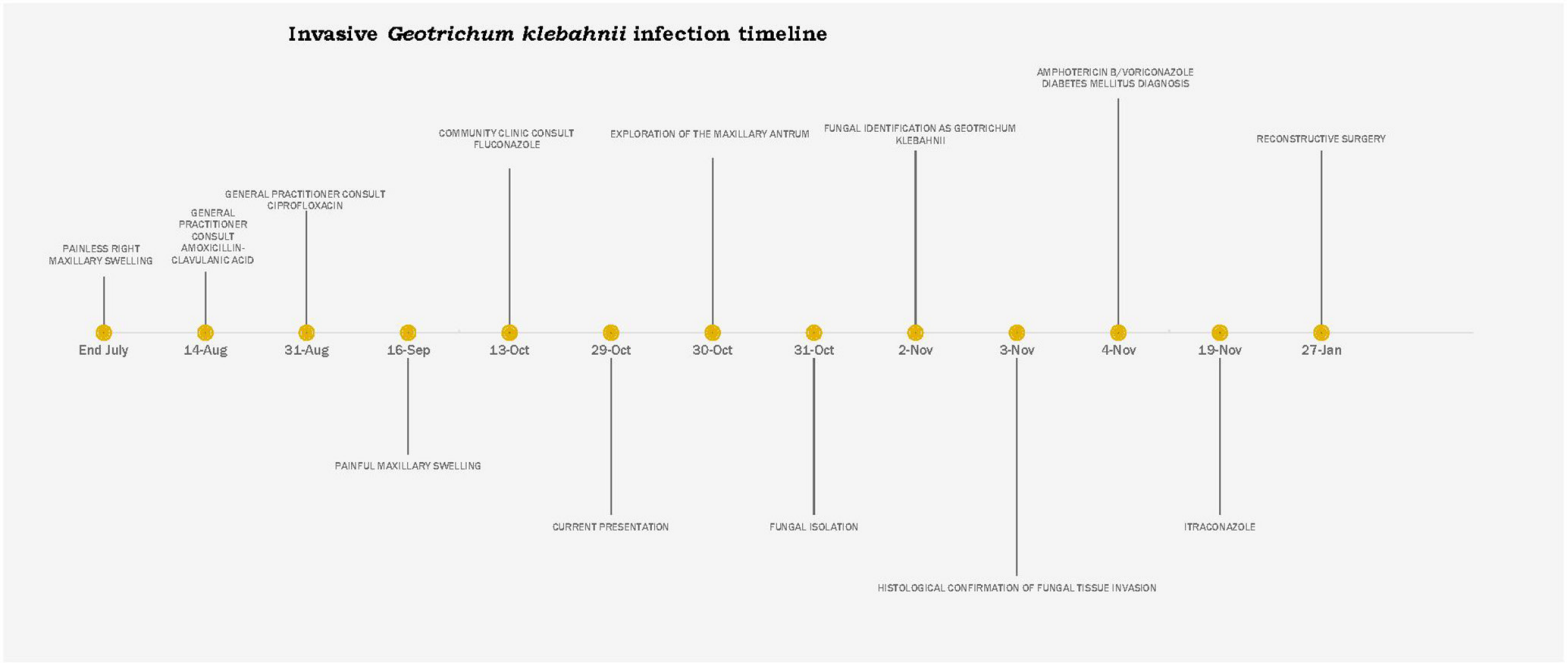
Timeline of events shown in weeks since first presentation of patient.

In view of the above findings, patient was switched from fluconazole to amphotericin B 1 mg kg^−1^ daily and voriconazole 400 mg 12 hourly for 24 h followed by 200 mg 12 hourly given for 2 weeks, followed by itraconazole 200 mg daily for 8 weeks (voriconazole was out of stock). The patient was also investigated for diabetes mellitus. Fasting blood glucose (219.6 mg dl^−1^) and HbA_1_C (18%) confirmed the diagnosis. This was treated with lifestyle modification and metformin. She was further referred for reconstructive surgery and made a full recovery. Refer to Fig. 3 for the timeline.

## Discussion

Invasive fungal infections involving non-*Candida* yeasts, thought to represent less than 5% of the yeast isolates, are emerging as causes of opportunistic infection with high morbidity and mortality in immunocompromised patients [19]. The incidence has been increasing in recent years as the spectrum of fungal infections has expanded due to an increase in the number of people living with immunocompromising conditions such as acquired immunodeficiency syndrome (AIDS) [19, 20]. The development of life-prolonging immunosuppressive agents for treatment of malignant and non-malignant diseases has also led to increased incidence [19]. These severely immunosuppressed patients are susceptible to infections from a variety of microorganisms including fungi that are rarely seen, or never reported as human pathogens [19]. *Geotrichum* spp. are some of these opportunistic fungi found in various environmental sources, as part of the ecosystem of healthy human skin, and frequently isolated from non-sterile samples from healthy individuals [3, 10–13, 19]. Most reported cases of geotrichosis involve *G. candidum*, *G. capitatus* and *G. clavatum* [1, 2, 4, 10, 14, 19]. The case presented here was due to infection with *G. klebahnii*. Similar to other *Geotrichum* spp., this fungus is ubiquitous in the environment worldwide but to the best of our knowledge there are no reported cases of human infection. Geotrichosis is almost always reported in an immunocompromised host [14, 15, 19] and our patient; who on presentation was not known to have an immunocompromising condition; was later confirmed to have had undiagnosed diabetes mellitus. The absence of diagnosis meant that the patient’s condition was not treated and hyperglycaemic remained uncontrolled as evident by the HbA_1_C of 18%. This underscores the importance of investigating patients for an immunocompromising condition whenever a diagnosis of geotrichosis is made.

Since *Geotrichum* spp. are saprophytic and ubiquitous in the environment, culture of the organism must always be correlated with clinical findings. Direct microscopic demonstration of the organism in clinical specimens and its repeated isolation in pure and luxuriant growth remain the gold standard of diagnosis of geotrichosis [4]. Our case had both culture of the organism and histological demonstration of tissue invasion by the pathogen. On Sabouraud dextrose agar, colonies are observed in 24 to 48 h, flat, off-white to cream, dry and finely suede-like with no reverse pigment [21]. *Geotrichum* species produce chains of hyaline, smooth, one-celled, subglobose to cylindrical, arthroconidia through holoarthric fragmentation of undifferentiated hyphae. The arthroconidia are variable in size and may germinate giving the appearance of a bud at one end. The genus does not produce true blastoconidia, a characteristic that distinguishes *Geotrichum* spp. from *Trichosporon* spp., which usually produce blastoconidia [22, 23]. These characteristics are shared by all species in the genus and it is impossible to speciate without doing further testing such as assimilation, sequencing or proteomic studies. *Geotrichum* species in our case was confirmed with assimilation tests and MALDI-TOF MS.

Species from the *Geotrichum* genus are often found to have low MICs to amphotericin B, itraconazole, posaconazole and flucytosine; while demonstrating elevated MICs to fluconazole [14, 23]. This is consistent with susceptibility profile in our case. Our patient also demonstrated a poor response to treatment with fluconazole and a positive response to amphotericin B and itraconazole. The echinocandins class of antifungals have unpredictable activity against *Geotrichum* spp., with reported MICs ranging from 0.06 µg ml^−1^ to >8 µg ml^−1^ [8, 14, 23]. Cases of treatment failure and breakthrough infections associated with echinocandin use have been reported [19]. The current treatment recommendations for geotrichosis exclude echinocandins as treatment options [14, 24]. The genus should be considered intrinsically resistant to echinocandins and the agents should not be used for the treatment of geotrichosis [14, 24].

## Conclusion

We described a rare case of invasive *G. klebahnii* infection in a patient with undiagnosed diabetes mellitus. *Geotrichum* spp. are rare emerging fungi which may be associated with a high morbidity and mortality. Immunocompromising conditions should be sought in individuals diagnosed with geotrichosis. Similarly, clinicians should have a high index of suspicion of geotrichosis in immunocompromised patient with lesions not adequately responding to treatment.
